# Effectiveness of an eHealth intervention for reducing psychological distress and increasing COVID-19 knowledge and protective behaviors among racialized sexual and gender minority adults: A quasi-experimental study (#SafeHandsSafeHearts)

**DOI:** 10.1371/journal.pone.0280710

**Published:** 2024-05-03

**Authors:** Peter A. Newman, Venkatesan Chakrapani, Notisha Massaquoi, Charmaine C. Williams, Wangari Tharao, Suchon Tepjan, Surachet Roungprakhon, Joelleann Forbes, Sarah Sebastian, Pakorn Akkakanjanasupar, Muna Aden

**Affiliations:** 1 Factor-Inwentash Faculty of Social Work, University of Toronto, Toronto, Ontario, Canada; 2 Centre for Sexuality and Health Research and Policy, Chennai, India; 3 Department of Health and Society, University of Toronto Scarborough, Scarborough, Toronto, Ontario, Canada; 4 Women’s Health in Women’s Hands Community Health Centre, Toronto, Ontario, Canada; 5 VOICES-Thailand Foundation, Chiang Mai, Thailand; 6 Faculty of Science and Technology, Rajamangala University of Technology, Phra Nakhon, Bangkok, Thailand; The Chinese University of Hong Kong, HONG KONG

## Abstract

**Purpose:**

Sexual and gender minority and racialized populations experienced heightened vulnerability during the Covid-19 pandemic. Marginalization due to structural homophobia, transphobia and racism, and resulting adverse social determinants of health that contribute to health disparities among these populations, were exacerbated by the Covid-19 pandemic and public health measures to control it. We developed and tested a tailored online intervention (#SafeHandsSafeHearts) to support racialized lesbian, gay, bisexual, transgender, queer, and other persons outside of heteronormative and cisgender identities (LGBTQ+) in Toronto, Canada during the pandemic.

**Methods:**

We used a quasi-experimental pre-test post-test design to evaluate the effectiveness of a 3-session, peer-delivered eHealth intervention in reducing psychological distress and increasing Covid-19 knowledge and protective behaviors. Individuals ≥18-years-old, resident in Toronto, and self-identified as sexual or gender minority were recruited online. Depressive and anxiety symptoms, and Covid-19 knowledge and protective behaviors were assessed at baseline, 2-weeks postintervention, and 2-months follow-up. We used generalized estimating equations and zero-truncated Poisson models to evaluate the effectiveness of the intervention on the four primary outcomes.

**Results:**

From March to November 2021, 202 participants (median age, 27 years [Interquartile range: 23–32]) were enrolled in #SafeHandsSafeHearts. Over half (54.5%, n = 110) identified as cisgender lesbian or bisexual women or women who have sex with women, 26.2% (n = 53) cisgender gay or bisexual men or men who have sex with men, and 19.3% (n = 39) transgender or nonbinary individuals. The majority (75.7%, n = 143) were Black and other racialized individuals. The intervention led to statistically significant reductions in the prevalence of clinically significant depressive (25.4% reduction, p < .01) and anxiety symptoms (16.6% reduction, p < .05), and increases in Covid-19 protective behaviors (4.9% increase, p < .05), from baseline to postintervention.

**Conclusion:**

We demonstrated the effectiveness of a brief, peer-delivered eHealth intervention for racialized LGBTQ+ communities in reducing psychological distress and increasing protective behaviors amid the Covid-19 pandemic. Implementation through community-based organizations by trained peer counselors supports feasibility, acceptability, and the importance of engaging racialized LGBTQ+ communities in pandemic response preparedness. This trial is registered with ClinicalTrials.gov, number NCT04870723.

## Introduction

Marginalization of lesbian, gay, bisexual, transgender, queer and other persons (LGBTQ+) outside of heteronormative and cisgender identities, and racialized people, exacerbates vulnerability to SARS-CoV2 transmission and serious Covid-19 outcomes. Marginalization refers to the processes through which certain populations are systematically excluded or relegated to the periphery of social, economic, and political participation and power [1, 2], reflected in differential access to and inequity in employment, education, housing, healthcare [1] and health information [3]. Adverse social determinants of health (SDOH) due to marginalization, both preceding and during the Covid-19 pandemic, contribute to excess risk among LGBTQ+ [4–7], Black [8] and other racialized populations [9, 10], and those with intersectional marginalized identities [6, 11]. In Canada, LGBTQ+ people are more likely to experience precarious employment and financial insecurity [12, 13], unstable housing [13], and lack of availability of competent healthcare [14] compared to cisgender heterosexuals—even more so among racialized LGBTQ+ populations and women [15]. These adverse SDOH increase risks for Covid-19 transmission, reduce access to care, and constrain the ability to adhere to public health-recommended nonpharmaceutical interventions, such as physical distancing, working from home, and Covid-19 testing.

The pervasive health and mental disparities resulting from marginalization of LGBTQ+ and racialized LGBTQ+ people that preceded Covid-19 also exacerbate vulnerability in the pandemic. Significantly higher prevalences of cancer, chronic kidney disease, chronic obstructive pulmonary disease (COPD), heart conditions, and type II diabetes mellitus among sexual minorities, and more so racialized sexual minorities, than among heterosexuals increase risks for more severe Covid-19 illness [11]. Moreover, lower levels of Covid-19 knowledge reported among Black communities than other groups [16, 17], a consequence of marginalization [3], increase pandemic-related risks.

Multiple pathways through which marginalization increases mental health risks among LGBTQ+ individuals include constrained access to financial and social resources, absence of LGBTQ+-competent healthcare, and sexual minority stress [18, 19]. Sexual minority stress is a unique form of stress experienced by LGBTQ+ individuals, in addition to general life stressors, owing to their sexual orientation in the absence of protective social policies and laws [18–20]. Compared to cisgender heterosexual individuals, gender and sexual minority individuals are disproportionately exposed to violence, harassment, discrimination, and social exclusion from families, peers, and communities, which contribute to sexual minority stress and disproportionate mental health problems, including depression and anxiety [18, 19, 21].

Thus, while the Covid-19 pandemic created stress and mental health challenges for much of the world’s population [22], ongoing marginalization and resulting mental health disparities among LGBTQ+ and racialized people are likely to exacerbate the trauma and social isolation of the pandemic [6, 23–25]. Evidence from Canada [26–28] and the U.S. [11, 29] indicates disproportionately higher rates of depression, anxiety, and loneliness among LGBTQ+ people compared to cisgender heterosexual individuals during the pandemic. Yet, despite documented inequities in SDOH, and resulting health and mental health disparities, which combine to increase pandemic-related risks among LGBTQ+ and racialized populations, we are aware of no tailored, evidence-informed interventions to reduce the burden of Covid-19 among these communities.

We developed and tested #SafeHandsSafeHearts, a peer-delivered eHealth intervention, with the aim of providing psychosocial and behavioral support and education to racialized LGBTQ+ individuals during the Covid-19 pandemic. The primary aims of the study were to test the effects of #SafeHandsSafeHearts on reducing clinically relevant depression symptoms and anxiety symptoms, and increasing Covid-19 knowledge and protective behaviors that mitigate the transmission of infection [30]. Our broader objective was to advance pandemic responses designed for racialized and LGBTQ+ populations.

## Materials and methods

All study procedures were approved by the Research Ethics Board of the University of Toronto (protocol no. 39769) and conducted in accordance with the Declaration of Helsinki. Informed consent was obtained online from all participants by clicking on “Accept” at the end of the online consent form before data collection took place. In case of any adverse events, responses followed the protocol of our lead community health center partner, with licensed, culturally competent medical and counseling professionals available in the event of participant distress or other emergency situations. The clinical trial was registered on ClinicalTrials.gov (NCT04870723). The study protocol has been published elsewhere [31]. Recruitment of participants for baseline, postintervention, and 2-month follow-up assessments lasted from March 1, 2021 to May 15, 2022. The study was registered prospectively as one site of an international randomized clinical trial with a crossover design; however, due to protracted delays in ethics approvals from the two international sites amid a public health emergency, we implemented the approved study at the Toronto site using a quasi-experimental pretest-posttest design. All enrollment criteria remained the same, and the intervention and assessments were conducted according to the registered protocol. The authors confirm that all ongoing and related trials for this intervention are registered. The supporting CONSORT checklist is available as supporting information; see S1 Checklist.

### Study design and setting

A quasi-experimental, single group pretest-posttest design was used to assess the effectiveness of the #SafeHandsSafeHearts eHealth intervention. We used a single group design, rather than a randomized clinical trial (RCT) with a control or waitlist group, due to practical and ethical considerations. For one, we followed a World Health Organization-recommended approach [32] which included community engagement in intervention development, co-governance, and capacity-building of community-based organizations (CBOs). Our primary CBO partner, a community health center, expressed a strong desire to provide the intervention to all individuals who enrolled in the study amid ongoing pandemic-related lockdowns in the study site. Secondly, given the disproportionate impact of the Covid-19 pandemic on the physical and mental health of our study population—LGBTQ+ and racialized people [6, 23, 24, 29]—we decided against a waitlist group, which would delay access to the intervention during a public health emergency.

The study was wholly developed and conducted online due to rolling lockdowns in Toronto and the contiguous urban region from March 2020 to June 2021 [33]. The Greater Toronto and Hamilton Area (GTHA; pop. estimate, 7.3 million) is comprised of the largest and most ethnoracially diverse cities by population in Ontario, including Toronto, Halton, Peel, York, and Durham.

### Sample size

The sample size was calculated based on power (80%) to detect significant differences (alpha = 0.05 for 95% confidence interval, two-tailed test) in 4 primary outcomes: increases in Covid-19 knowledge and Covid-19-protective behavior scores; and decreases in the proportion of participants with pandemic-related depressive and anxiety symptoms. We assumed a small to medium effect size (Cohen’s d = 0.3) on knowledge and behavior, and 20% reduction in the proportion of participants with depression or anxiety, based on an extensive review of health behavior and stress management interventions [34]. Required sample sizes estimated using Stata-16 and G*Power 3.1 (McNemar’s paired-proportions test) ranged from 90–92 to detect significant differences between pre-intervention and postintervention timepoints [35]. Assuming 10% attrition and adding a design/clustering effect of 1.5, the target sample size was increased to 239.

### Procedures

We developed a customized online dashboard and database to support study coordination, including tracking of participant recruitment, screening, enrollment, counselor assignment, and eHealth sessions. Participants were recruited online via listservs and social media accounts of CBOs and health centers serving racialized and LGBTQ+ communities, LGBTQ+ e-groups, and a study website. We distributed e-flyers and messages with a focus on groups and organizations serving racialized LGBTQ+ populations.

Eligibility criteria were (1) age ≥18 years; (2) self-identify as cisgender lesbian or bisexual woman or woman who has sex with women (LBWSW); cisgender gay or bisexual man or man who have sex with men (GBMSM); or transgender or non-binary individuals (TNB); (3) resident in the GTHA for ≥ 6 months; (4) able to understand and willing to provide informed consent; and (5) able to understand English.

Peer counselors who delivered the intervention were MSW student interns in health and mental health specializations and BSW-level counselors from CBOs that provide services to sexual and gender minority populations. All had previous experience working with racialized communities. Most peer counselors identified as racialized, and all were LGBTQ+ or allies. Peer counselors completed a university-accredited online training course in motivational interviewing (MI) before delivering the intervention. Peer counselors also completed a 3-day, manualized online training delivered by health and mental health professionals on the conduct of the intervention, including small-group discussions, role-plays, mock counseling sessions, and feedback, as well as a subsequent booster session. The training covered Covid-19, public health-recommended nonpharmaceutical interventions, pandemic-related distress (i.e., anxiety, depression, social isolation), MI-based counseling, psychoeducation, and research ethics. In addition, two Covid-19 medical updates were provided by medical officers from Toronto Public Health. Throughout the intervention, an experienced, licensed social worker conducted biweekly clinical group supervision including peer counselor self-assessments and supervisor feedback. Peer counselors also completed brief self-assessments online after each session was conducted. MSW student interns were not paid as they were gaining course credit through their university. Community counselors were paid $30 per hour.

### Intervention

#SafeHandsSafeHearts builds on evidence-informed interventions that have used MI [36, 37] and psychoeducation [38] to increase health knowledge, health behaviors, and reduce psychological distress [39–41]. As a client-centered, nonjudgmental, and strengths-based approach [42, 43], MI is particularly appropriate for working with individuals from marginalized populations who have historically been subjected to stigma and directed to conform to hetero- and cis-normative and dominant ethnocultural expectations and behaviors [44, 45]. MI shifts the locus of control from the counselor to the client and promotes resilience by strengthening intrinsic motivation for change [45, 46]. Consonant with the tenets of MI, psychoeducation is a strengths-based approach that integrates education and counseling, in which clients are considered partners in treatment [38].

MI has demonstrated effectiveness in health behavior change among sexual minorities, though interventions largely focus on gay White men and HIV risk reduction and substance use behaviors [15, 47, 48]. Overall reviews of the effectiveness of MI have indicated limited research with racialized populations and mixed results [43, 46]; however, one recent review focused on MI-based interventions with racialized populations identified acknowledgement of participants’ culture and cultural tailoring of the intervention, as well as counselors being of similar backgrounds to participants, as hallmarks of success [44]. Informed by elements of effective MI interventions and the importance of cultural tailoring, rather than adapt previous interventions designed for cisgender gay and predominantly White men for implementation with racialized cisgender women and transgender people, we marshalled the multidisciplinary expertise, clinical and research experience, and cultural and sexual/gender diversity of our research team and community partners to design an intervention tailored for racialized sexual and gender minority individuals.

The intervention content was organized into 3 sessions, based on systematic reviews of MI-based interventions for health-related behaviors [41, 46] (see S1 Appendix). The first session focused on building rapport, goal-identification (i.e., participant’s goals for change), and psychoeducation (i.e., selecting 1 or 2 new things to observe/try out and discuss next session), with additional content focused on knowledge about Covid-19 transmission, symptoms, testing, and treatment. Session 2 content focused on understanding and practicing Covid-19 protective behaviors (i.e., improving self-efficacy), risk reduction, psychoeducation, and problem-solving (i.e., reviewing the “new things” experience, reinforcing successes, normalizing setbacks). Session 3 focused on understanding psychosocial issues, promoting awareness of community resources, and improving mental health (i.e., mental health and social support assessment and strengthening, expanding support for change—tools, relationships, services), and maintaining change (i.e., relapse prevention).

The intervention was delivered online (via mobile phone, tablet, laptop, or PC) in 3-weekly 60-minute modules. Counselors were given flexibility to address pressing needs raised by participants at the beginning of each session. A referral list was provided to all counselors, including locally available and free or low-cost concrete services (e.g., food banks, housing), health services, and mental health hotlines, including LGBTQ+- and ethnoracially-competent organizations. Counselors documented referrals made in each session and followed up with participants in subsequent sessions. Participants were provided with a $30 honorarium after each online session, and after postintervention and follow-up survey completion.

### Measures

Participants completed surveys at baseline, 2-weeks postintervention after their final eHealth session, and 2-month follow-up.

#### Primary outcomes

Depression symptoms in the past 2 weeks were measured using the Patient Health Questionnaire-2 (PHQ-2) [49]. Anxiety symptoms in the past 2 weeks were measured using the Generalized Anxiety Disorder-2 scale (GAD-2) [50]. Each 2-item scale was scored from 0–3 (range, 0–6), with a score of ≥3 indicative of screening cut-points for clinically significant depression or anxiety [51].

Covid-19 knowledge was assessed using a 7-item index (score range, 2–7) developed by the research team, based on U.S. Centers for Disease Control (CDC) guidelines [52, 53] and published research [54, 55]. Public health-recommended Covid-19 protective behaviors (handwashing, mask-wearing, physical distancing) were assessed using a 9-item index (score range, 1–18) developed by the research team based on WHO and U.S. CDC guidelines [52, 53].

#### Demographic characteristics and other covariates

Demographic variables included age, sex, gender identity, sexual orientation, race/ethnicity, city of residence, country of birth, education, and employment status. We assessed loneliness/social isolation (UCLA Loneliness Scale) [56]; Covid-19 stress (Covid-19 danger and Covid-19 traumatic stress subscales) [57]; resilience (Resilience to Traumatic Experience scale) [58]; vaccine conspiracy beliefs [59–61]; and Covid-19 vaccination status.

### Acceptability of the intervention

In order to maximize the use and efficiency of the research developed and conducted during a real-world public health emergency, we included a brief assessment of the acceptability of the eHealth intervention [62]. This responded to our community partner’s interest in developing their own eHealth programming, and the research team’s plans to conduct an RCT in two additional sites. After each eHealth session, participants were sent an online link to a confidential 4-item evaluation to indicate their satisfaction with the session and session length, the extent to which it was helpful in improving their emotional wellness or mental health and their Covid-19-related knowledge and skills.

### Statistical analyses

Descriptive statistics were used to summarize sociodemographic and related characteristics: frequencies and percentages for categorical variables and means and standard deviations for continuous variables. We used population-averaged Poisson models (generalized estimating equations or GEE) [63] to estimate dichotomous outcomes (i.e., depression, anxiety), and zero-truncated Poisson models [64] to estimate count outcomes (i.e., Covid-19 knowledge, Covid-19 protective behaviors). Incidence rate ratios (IRR) were calculated based on pair-wise comparisons, with Bonferroni’s method used for multiplicity testing adjustments, to estimate between group differences on primary outcomes at baseline, postintervention, and 2-month follow-up. Clustering of observations at the participant level were taken into account by specifying the clustering variable (participant ID) in the GEE models and by using robust standard errors in the truncated Poisson models [65]. We conducted an intention-to-treat analysis, in that all participants were included irrespective of their session attendance or completion of questionnaires. A complete-case analysis is generally not recommended due to loss of sample size and potential bias in the results [66]. Given the quasi-experimental design of the study and potential confounding effects of certain sociodemographic characteristics on study outcomes, models were adjusted for age [67, 68], race/ethnicity [69], sexual/gender identity [29, 67, 68], education [67] and employment status [70]. Two-sided p-values < 0.05 were considered statistically significant. All analyses were performed using Stata/SE 16.1 (Stata Corporation, College Station, TX).

## Results

### Enrollment and intervention exposure

**Fig 1:**
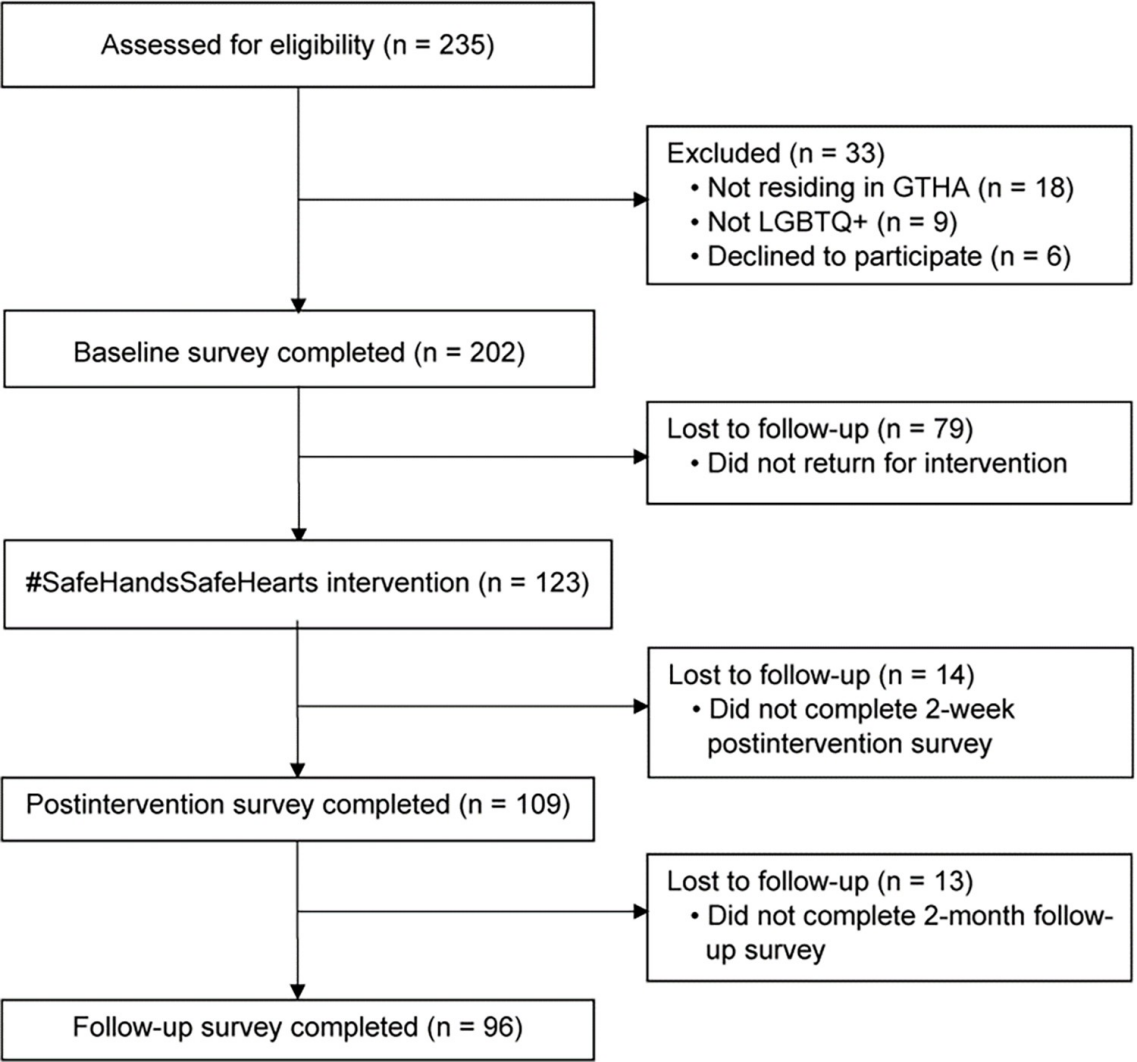
CONSORT diagram of participant flow in one-group quasi-experimental pretest-posttest design (#SafeHandsSafeHearts). GTHA, Greater Toronto and Hamilton Area; LGBTQ+, lesbian, gay, bisexual, transgender, queer, and other persons outside of heteronormative and cisgender identities.

Among individuals initially screened online (n = 229), 27 (11.8%) were ineligible; of these, n = 18 did not meet residency requirements and n = 9 did not meet sexuality/gender criteria. Among enrolled participants (n = 202), the majority (54.5%; n = 110) completed 3 sessions, 5 (2.5%) completed 2 sessions, 8 (4.0%) 1 session, and 79 (39.1%) no sessions. Fifty-four percent (n = 109) completed the postintervention survey, and 48% (n = 96) completed the follow-up survey (see Fig 1).

### Participant characteristics

In total, 202 participants (median age, 27 years [IQR: 23–32]) were enrolled in the #SafeHandsSafeHearts trial. The majority (54%, n = 110) identified as cisgender LBWSW, nearly one-fifth (19.3%, n = 39) TNB, and one-quarter (26.2%, n = 53) cisgender GBMSM. Nearly one-third of participants (30.7%, n = 62) identified as African/Caribbean/Black, 30.2% (n = 61) South/East/Southeast Asian, 24.3% (n = 49) White, 8.9% (n = 18) Latinx/Hispanic, and 5.9% (n = 12) multiracial. Over two-thirds (67.3%, n = 136) had college-degree education and one-half (50.0%, n = 101) were unemployed. Table 1 shows participant demographic characteristics.

**Table 1:** Baseline demographic characteristics (N = 202).

Characteristic	n (%)
**Race/ethnicity**	
African/Caribbean/Black	62 (30.7)
South/East/Southeast Asian	61 (30.2)
Latinx/Hispanic	18 (8.9)
White	49 (24.3)
Multiracial	12 (5.9)
**Gender**	
Cisgender lesbian, bisexual, WSW	110 (54.5)
Cisgender gay, bisexual, MSM	53 (26.2)
Transgender or nonbinary	39 (19.3)
**Education**	
None/Elementary school	6 (3.0)
High school	60 (29.7)
≥ College/University	136 (67.3)
**Employment**	
Full-time	47 (23.3)
Part-time	54 (26.7)
Unemployed	101 (50.0)
**Health Insurance**	
Insured	164 (81.2)
Uninsured	38 (18.8)

MSM, men who have sex with men; WSW, women who have sex with women

### Psychological distress, Covid-19 knowledge and protective behaviors

The baseline prevalence of depressive symptoms was 53.6% (95% CI 48.0%–59.3%), and of anxiety symptoms was 63.5% (95% CI 57.5%–69.5%). The mean score on Covid-19 knowledge at baseline was 6.73 (95% CI 6.66–6.80) and on protective behaviors was 14.75 (95% CI 14.27–15.23) (see Table 2).

**Table 2:** Effect of the intervention on primary outcomes: Predicted probabilities (%) or scores, and pairwise comparisons of change in % or score at 3 timepoints.

Outcomes	Scores or percentages at 3 timepoints			Pairwise comparisons		
	(95% CI)			IRR (95% CI)		
	*Baseline*	*Post-intervention*	*Follow-up*	T0 to T1	T0 to T2	T1 to T2
	*(T0)*	*(T1)*	*(T2)*			
**Depressive symptoms (%)**	53.6%	40.0%	49.1%	0.746**^a^	0.915	1.225
	(48.0, 59.3)	(32.4, 47.7)	(39.2, 58.9)	(0.612–0.911)	(0.737–1.135)	(0.951–1.579)
**Anxiety symptoms (%)**	63.5%	52.9%	48.4%	0.834*	0.763*	0.915
	(57.5, 69.5)	(44.5, 61.3)	(38.8, 58.0)	(0.710–0.979)	(0.615–0.945)	(0.732–1.144)
**Covid-19 knowledge score (2–7)**	6.73	6.68	6.67	0.992	0.991	0.998
	(6.66, 6.80)	(6.57, 6.80)	(6.52, 6.82)	(0.973–1.013)	(0.967–1.016)	(0.970–1.027)
**Covid-19 protective behaviors score (1–18)**	14.75	15.48	16.02	1.049*^b^	1.086***	1.035
	(14.27, 15.23)	(15.02, 15.94)	(15.55, 16.49)	(1.002–1.099)	(1.036–1.138)	(0.994–1.078)

*p < .05

**p < .01

***p < .001

^a^ IRRs can be interpreted as relative differences; an IRR of 0.746 signifies a 25.4% decrease in the prevalence of depression from baseline (T0) to postintervention (T1) (i.e., 0.746–1 = -.254 or -25.4%)

^b^ This signifies a 4.9% increase in the mean score of Covid-19 protective behaviors from baseline to postintervention (1.049–1 = .049 or 4.9%)

CI, confidence interval; IRR, incidence rate ratio

### Effect of the intervention on depression

The intervention led to statistically significant reductions in the prevalence of depressive symptoms by one-fourth (25.4%) from baseline to postintervention (IRR = 0.746; 95% CI 0.612–0.911, p < .01) (Table 2). Those who were employed reported significantly lower risk (IRR = 0.74; 95% CI .60–.90, p < .05) of depressive symptoms (Table 3). Higher scores on Covid-19 stress (IRR = 1.25; 95% CI, 1.13–1.39, p < .001) and loneliness (IRR = 1.24; 95% CI 1.16–1.32, p < .001) were associated with significantly greater risks of depressive symptoms (Table 2). Higher scores on resilience to traumatic stress were associated with a small but significantly lower risk of depressive symptoms (IRR = 0.97; 95% CI .94–.99, p < .001). Overall reduction in the prevalence of depression from baseline to 2-month follow-up was not statistically significant, nor was the difference between postintervention and follow-up prevalence of depressive symptoms statistically significant, suggesting that the effect of the intervention on depression was not retained.

**Table 3:** Predictors of depressive and anxiety symptoms among LGBTQ+ individuals in Toronto (n = 202).

	Depression			Anxiety		
Variables	IRR	SE	95% CI	IRR	SE	95% CI
**Time** (Ref. Baseline)						
Postintervention	0.75**	0.08	0.61–0.91	0.83*	0.07	0.71–0.98
Follow-up	0.91	0.10	0.74–1.13	0.76*	0.08	0.62–0.94
**Age**	0.99	0.00	0.98–1.00	1.00	0.00	0.99–1.01
**Employed** (Yes)	0.74**	0.07	0.61–0.90	0.84*	0.07	0.70–0.99
**Identity** (Ref. Cisgender GBMSM)						
Cisgender LBWSW	1.23	0.17	0.93–1.62	1.29*	0.16	1.01–1.66
Transgender/Nonbinary	1.14	0.18	0.84–1.55	1.21	0.18	0.91–1.62
**Education**: College (Ref. Up to higher secondary)	1.00	0.10	0.83–1.21	1.04	0.10	0.86–1.25
**Covid-19 stress score**	1.25***	0.07	1.13–1.39	1.29***	0.07	1.16–1.43
**Loneliness score**	1.24***	0.04	1.16–1.32	1.14***	0.03	1.08–1.21
**Resilience to traumatic experience score**	0.97**	0.01	0.94–0.99	1.00	0.01	0.98–1.02
**Race** (Ref. White)						
African/Caribbean/Black	1.20	0.15	0.94–1.52	0.90	0.10	0.72–1.13
South/East/SE Asian	0.89	0.12	0.69–1.16	0.91	0.10	0.72–1.14
Latinx/Hispanic	1.11	0.27	0.69–1.79	1.18	0.21	0.84–1.66
Multiracial	1.16	0.16	0.89–1.51	1.17	0.19	0.85–1.61
**Constant**	0.15***	0.06	0.07–0.33	0.13***	0.05	0.07–0.26

*p < .05

**p < .01

***p < .001

SE, standard error

GBMSM, gay, bisexual and men who have sex with men; LBWSW, lesbian, bisexual women who have sex with women; SE Asian, Southeast Asian

### Effect of the intervention on anxiety

The intervention led to statistically significant reductions in anxiety symptoms, with the prevalence reduced by 16.6% from baseline to postintervention (IRR = 0.834; 95% CI 0.710–0.970, p < .05) (Table 2). The prevalence of anxiety symptoms at 2-month follow-up was also significantly different from baseline (23.7% reduction: IRR = 0.763; 95% CI 0.615–0.945, p < .05), with no significant difference from postintervention to follow-up (IRR = 0.915; 95% CI 0.732–1.144, p = .65); this suggests that the effect of the intervention on reducing anxiety was retained over time (Table 2). Those who were employed had a significantly lower risk (IRR = 0.84; 95% CI, .70–.99, p < .05) of anxiety symptoms (Table 3). Cisgender sexual minority women had a significantly higher risk (IRR = 1.29; 95% CI 1.01–1.66, p < .05) of anxiety symptoms compared to cisgender sexual minority men. Higher scores on Covid-19 stress (IRR = 1.29; 95% CI 1.16–1.43, p < .001) and higher scores on loneliness (IRR = 1.14; 95% CI 1.08–1.21, p < .001) were each associated with significantly greater risk of anxiety symptoms.

### Effect of the intervention on Covid-19 knowledge

There was no statistically significant increase in Covid-19 knowledge scores over time (Table 2). Higher scores on vaccine conspiracy beliefs (IRR = 0.994; 95% CI, 0.990–0.998, p < .05) were inversely associated with Covid-19 knowledge scores over time (Table 4). Higher Covid-19 stress scores were positively associated with Covid-19 knowledge (IRR = 1.02; 95% CI, 1.005–1.04, p < .05) over time.

**Table 4:** Predictors of Covid-19 knowledge and protective behavior scores in Toronto (n = 202).

	Covid-19 Knowledge Score			Covid-19 Behavior Score		
Variables	IRR	SE	95% CI	IRR	SE	95% CI
**Time** (Ref. Baseline)						
Postintervention	0.99	0.01	0.97–1.01	1.05*	0.02	1.00–1.10
Follow-up	0.99	0.01	0.97–1.02	1.09***	0.03	1.04–1.14
**Age**	1.00	0.00	1.00–1.00	1.00	0.00	1.00–1.00
**Employed** (Yes)	0.99	0.01	0.97–1.01	1.03	0.02	0.99–1.07
**Identity** (Ref. Cisgender GBMSM)						
Cisgender LBWSW	1.00	0.01	0.98–1.02	1.03	0.03	0.98–1.09
Transgender/Nonbinary	1.01	0.01	0.98–1.03	1.02	0.04	0.95–1.10
**Education**: College (Ref. up to higher secondary)	1.02*	0.01	1.00–1.04	0.97	0.02	0.93–1.02
**Vaccine conspiracy beliefs score**	0.99**	0.00	0.990–0.998	1.00	0.00	0.99–1.00
**Covid-19 stress score**	1.02*	0.01	1.005–1.04	1.03*	0.01	1.00–1.05
**Covid-19 knowledge score**				1.06***	0.02	1.03–1.10
**Vaccinated with ≥1 dose** (Yes)				0.93*	0.03	0.89–0.99
**Race** (Ref. White)						
African/Caribbean/Black	1.02	0.01	1.00–1.05	1.06*	0.03	1.00–1.12
South/East/SE Asian	1.02*	0.01	1.00–1.05	1.05*	0.03	1.00–1.11
Latinx/Hispanic	1.04**	0.02	1.01–1.07	1.02	0.05	0.93–1.13
Multiracial	0.99	0.03	0.95–1.04	1.08*	0.04	1.02–1.15
**Vaccinated with ≥1 dose** (Yes)	1.01	0.01	0.98–1.03			
**Constant**	6.58***	0.17	6.25–6.92	9.01***	1.27	6.84–11.87

*p < .05

**p < .01

***p < .001

### Effect of the intervention on Covid-19 protective behaviors

The intervention led to a statistically significant increase in Covid-19 protective behavior scores over time, from baseline to postintervention (4.9% increase: IRR = 1.049; 95% CI 1.002–1.099, p < .05), and from baseline to 2-month follow-up (8.6% increase: IRR = 1.086; 95% CI 1.036–1.138, p < .001) (Table 2). There was no significant change in Covid-19 protective behavior scores from postintervention to 2-month follow-up (IRR = 1.035; 95% CI 0.994–1.078, p = .06), suggesting that the effect of the intervention was retained. Those with higher Covid-19 knowledge scores showed small but statistically significant differences in higher scores on Covid-19 protective behaviors (IRR = 1.06; 95% CI 1.03–1.10, p < .001) over time (Table 4). Those who reported Covid-19 vaccination (one or two doses) showed small but statistically significant differences in lower scores on Covid-19 protective behaviors (IRR = 0.93; 95% CI 0.89–0.99, p < .05) over time.

### Acceptability of the intervention

Evaluation forms were submitted online by 72% of those who engaged in intervention counselling sessions. Overall, participants indicated feeling “very satisfied” with the session in 84% (141/168) of sessions evaluated and “very satisfied” with the duration of the session in 79% (133/168) of sessions evaluated. In 86% (145/168) of sessions evaluated, participants indicated they were “very helpful” in “improving your emotional wellness or mental health.”

## Discussion

This study reports the results of a novel community-based, peer-delivered eHealth intervention (#SafeHandsSafeHearts), which is to our knowledge the first intervention to demonstrate effectiveness in reducing pandemic-related psychological distress and increasing Covid-19 protective behaviors among LGBTQ+ people. Despite the largescale failure of public health systems to systematically report sexual orientation and gender identity information in relation to Covid-19 [71], increasing evidence indicates the disproportionate impact of Covid-19 and pandemic-related public health responses on health and mental health outcomes among LGBTQ+ people in Canada [26], the US [72, 73], and Western Europe [74].

Importantly, even among research and interventions designed to support broader LGBTQ+ health, there is substantially lesser focus on individuals who occupy intersectional marginalized identities based on sexual or gender minority and racialized status, such as Black, Latinx, Asian, and other racialized LGBTQ+ individuals [15, 24, 71]. Our successful enrollment of a sample of LGBTQ+ people, a majority of whom were racialized, is both notable and apropos of the elevated risk of Covid-19 infection and more severe outcomes among these populations [11]. The successful implementation of #SafeHandsSafeHearts during a global public health emergency, with extensive rolling lockdowns and stay-at-home orders in the Greater Toronto and Hamilton Area [33], further supports the feasibility and ecological validity of the intervention.

As hypothesized, the intervention led to a statistically significant reduction in the prevalence of symptoms indicative of clinically significant depression and anxiety from baseline to postintervention. The high baseline prevalence of depressive and anxiety symptoms itself exemplifies the extreme pandemic-related distress among racialized LGBTQ+ individuals, many of whom were enrolled before Covid-19 vaccination was available to them beginning in June 2021 [75].

The significant effects of the intervention in reducing the prevalence of depression and anxiety support the success of the MI-based and psychoeducational approach, as well as the eHealth modality. The decidedly nonjudgmental stance of MI may be especially appropriate in working with sexual and gender minority and racialized communities, more so amid a pandemic. These communities may understandably experience ambivalence or alienation in the ongoing context of structural racism [76, 77], transphobia and homophobia [71], and historically justified medical mistrust [78, 79], in response to public health interventions that were not designed with community input nor with their communities in mind [78, 80]. The use of eHealth, as well as telehealth and other online modalities with low barriers to access, also may be particularly appropriate for LGBTQ+, racialized, and other marginalized populations during a pandemic; closures of familiar community-based services may reduce access to culturally competent health and mental health services despite their heightened vulnerability [74]. Nevertheless, even with decreases in depressive symptomology 2-weeks post-intervention, the failure to retain this effect at 2-month follow-up suggests that future trials should assess the intervention with more than 3 sessions, and/or the use of eHealth booster sessions [81] to determine if any of these may support retention of reductions in depression over time. Although challenges have been reported for brief MI-based interventions in their effects waning over time [46], in the present study the retention of intervention effects in reducing anxiety at 2-months post-intervention during an ongoing pandemic suggests the potential for a more sustained impact on reducing depression.

We also identified statistically significant associations between secondary measures of loneliness and Covid-19 stress, respectively, and depression and anxiety outcomes. The intervention may have helped to abate these risk factors, potentially reflecting benefits of a peer-based approach in providing psychosocial support for marginalized communities in the context of systemic stigma and discrimination [82]. LGBTQ+ individuals may be particularly vulnerable to loneliness and psychological distress during prolonged lockdowns as a result of different family configurations than heterosexual individuals on whom public health responses are normed, and the closure of community spaces that provide LGBTQ+-affirmative and culturally competent support [83, 84].

Notably, employment exerted significant protective effects against both depression and anxiety; this corroborates pathways through which economic marginalization contributes to vulnerability in a pandemic [27, 71]. The extensive job loss reported among our sample is substantiated by Toronto government data indicating 50% higher rates of pandemic-related unemployment among Black and Asian versus White and other ethnoracial groups [85], as well as studies demonstrating vulnerability and stress associated with pandemic-related job loss among LGBTQ+ individuals in Canada [27] and the U.S. [29, 71]. Structural interventions to promote job security and retention among racialized people and sexual and gender minorities, communities disproportionately represented in service industries [6], such as provision of paid sick leave [86] and broader employment antidiscrimination measures, may exert substantial salubrious effects on mental health in a public health emergency.

Our findings also identify relatively high adherence to public health-recommended protective behaviors at baseline, including physical distancing and mask-wearing, as well as handwashing. The intervention had a small but sustained positive effect on protective behaviors, with increases retained at 2-month follow-up. The uniformly high Covid-19 knowledge demonstrated at baseline may be attributable to daily public health messaging about Covid-19 delivered through multiple channels in the Toronto area; this may have impeded our ability to detect improvements in Covid-19 knowledge over time.

### Strengths and limitations

Study results should be understood in the context of limitations. First, the quasi-experimental pretest-posttest design precludes the ability to determine causal associations; however, the temporality of the intervention, and use of pretest, posttest, and follow-up surveys, support the impact of the intervention on the outcomes assessed and account for possible decay in intervention effects over time [87]. Nevertheless, the 2-month follow-up time frame limits our ability to assess longer term intervention effects. Second, self-reporting of depression and anxiety symptoms, and use of a brief assessment instrument, might result in bias; however, we implemented widely used screening tools with established validity and reliability [51]. Third, while over 60% of individuals who screened into the study completed 1 or more—89% of these, all 3—counseling sessions, session nonattendance may reflect the challenges of participation and retention during a pandemic. We anticipated constraints to engaging in online counseling owing to lack of privacy, and not having a personal mobile device or broadband internet access. To that end, we made arrangements with local CBOs to provide free PC/internet access on site; however, ongoing lockdowns and concerns about infection may have precluded their utilization. The lack of pilot testing also limited our ability to assess the feasibility of the intervention and enact possible changes to reduce barriers to participation; however, we decided to forego pilot testing to avoid further delays in study implementation among vulnerable populations during a public health emergency. Nevertheless, even with the inclusion of participants who did not attend one or more sessions in our analyses, we identified intervention effectiveness on three of four primary outcomes.

Fourth, lack of retention of significant reductions in depression, similarly identified in other brief MI-based interventions [46], suggests the need to test #SafeHandsSafeHearts with additional eHealth sessions over a longer duration. Fifth, we solicited and received feedback on the intervention from professionals in social work, psychology, and medicine, however the intervention was not formally reviewed by an external professional expert panel. Finally, the study was conducted in one Canadian metropolitan area, and results may not be generalizable to all racialized LGBTQ+ people in Canada or elsewhere. However, we designed eligibility criteria to include a broad range of self-identifications among racialized sexual and gender minorities (i.e., not only self-identified gay or lesbian or trans individuals) and we were successful in recruitment of a predominantly racialized LGBTQ+ sample during a pandemic.

### Implications for research and practice

The present results suggest that future research should be conducted with larger sample sizes of racialized sexual and gender minority populations in other locales, and using randomized designs, to further validate the effectiveness of the #SafeHandsSafeHearts intervention. Our results also suggest augmenting the 3-session eHealth intervention with additional sessions, possibly with less frequency (every two weeks rather than weekly), and conducting a postintervention survey after a longer (i.e., 6-month) interval to evaluate effectiveness in sustaining reductions in psychological distress over time. As the intervention was developed and culturally tailored for racialized and LGBTQ+ communities in Toronto, Canada, and delivered by trained peers from within these communities and allies, pilot testing and community-engaged approaches in partnership with culturally appropriate organizations should be used to adapt and deliver the intervention for other communities and contexts [44].

In Canada as in other jurisdictions, LGBTQ+ communities, and more so ethnic/racial minority LGBTQ+ people, are likely to experience heightened vulnerability in future pandemics and emergency situations to the extent public health responses continue to be reflexively designed and normed on cisgender and heterosexual populations [31, 88, 89]. Implicit assumptions about living arrangements with two-parent families or cohabitating married couples, and the presumed availability of supportive family networks, may further disenfranchise LGBTQ+ individuals, whose lives may not correspond to these heteronormative expectations [6, 31]. In this context, public health measures to control the pandemic, such as protracted stay-at-home mandates, closures of local community organizations and healthcare services, and increased police surveillance, may exert greater psychosocial and financial stress on LGBTQ+ than on cisgender heterosexual individuals [6, 31]. The disparate impacts of Covid-19 and future pandemics on LGBTQ+ communities are also likely to be exacerbated in jurisdictions without LGBTQ+ human rights protections, particularly in contexts of criminalization [88, 90], and absent legal recognition of same-sex marriage [91].

Overall, the present study suggests the core importance of meaningful engagement of ethnoracially diverse LGBTQ+ and other marginalized communities and local CBOs in future research, intervention design and delivery [92], and the critical need for data inclusive of sexual and gender minorities [89], to better prepare for future health crises. Specifically, evidence from this study supports the need for multicomponent culturally tailored pandemic preparedness and interventions: LGBTQ+ affirmative eHealth and telehealth interventions to support mental health, coping, and sexual health [31, 80]; programs and policies to promote employment access and security [15, 70, 93]; ensuring access to LGBTQ+ competent and equitable healthcare [14, 70, 90]; and broader structural interventions to implement and enforce LGBTQ+ human rights protections and to combat intersectional stigma, discrimination, and violence against ethnoracially diverse sexual and gender minorities [14, 15, 31, 88, 90, 93].

## Conclusion

We demonstrated the preliminary effectiveness of an innovative, brief eHealth intervention in reducing psychological distress and increasing protective behaviors among LGBTQ+, predominantly racialized, individuals during the Covid-19 pandemic. Overall, this study affirms the critical importance of meaningful engagement of racialized LGBTQ+ communities in pandemic preparedness, and public health intervention design and implementation.

## Supporting information

S1 Checklist CONSORT 2010 checklist of information to include when reporting a randomised trial. (DOC)

S1 Appendix #SafeHandsSafeHearts intervention. (DOCX)
