# Supplementary material for: Effectiveness of an eHealth intervention for reducing psychological distress and increasing COVID-19 knowledge and protective behaviors among racialized sexual and gender minority adults: A quasi-experimental study (#SafeHandsSafeHearts)
Source: PLoS One. 2024 May 3;19(5):e0280710. doi: 10.1371/journal.pone.0280710 (PMC11068205; doi:10.1371/journal.pone.0280710)
Supplement: S1 Appendix — (DOCX) [file pone.0280710.s002.docx]

**S2. Appendix**

**#SafeHandsSafeHearts Intervention**

| **Online peer-delivered MI-based psychoeducation intervention** | | |
| --- | --- | --- |
| **#** | **Focal Content Description** | **Intervention Component** |
| **1** | **COVID-19 knowledge** | **Building rapport, goal-identification, psychoeducation** |
|  | - What is COVID-19 and the novel-Coronavirus? - Modes of infection, symptoms, diagnosis - Treatment and prognosis - Myths and misconceptions - Recovery, reactivation, reinfection - Initial mental health screening and social support assessment | - Establish helping relationship - Overview of intervention - Self-appraisal of COVID-19 knowledge - Enhancement of knowledge, identifying and dispelling myths and misconceptions - Identifying and normalizing COVID-19 effects on life, including mental health - Exploring client’s goals for change – areas, motivation, goals - Selecting 1 or 2 “new things” to observe/try out and discuss in the next session |
| 2 | **Assessing risk for infection & understanding and practising public health recommended protective behaviors** | **Understanding & practising public health recommended protective behaviors: risk education/risk reduction, psychoeducation, and problem-solving** |
|  | - Handwashing, wearing mask, physical distancing, self-isolation, self-quarantine, - What to do if one shows signs/symptoms, - Measures to be adopted if one gets infected - Mental health screening and assessment (anxiety, depression, social isolation) | - Overview of session - Discussing participant perspective on protective behaviours - Improving self-efficacy in protective behaviours - Identifying and addressing challenges in adhering to protective behaviours - Screening and providing counselling for mental health issues - Reviewing the ‘new things’ experience – reinforcing successes, normalizing setbacks, barriers, problem solving, including mental health and social support - Reviewing/revising goals and plans - Selecting new strategies to try out and discuss next session |
| **3** | **Understanding psychosocial issues and maintaining mental health** | **Risk reduction, psychoeducation,**  **maintaining change (relapse prevention)** |
|  | - Anxiety, depression, social isolation - Problematic alcohol and drug use screening - Develop tailored plan to maintain mental health - Assess available social support resources - Assist in getting support, building support systems | - Overview of session - Review of progress – changes, skills, awareness, attitudes, knowledge, support, problem-solving - Screening and providing counselling for mental health issues - Social support assessment - Assistance in social support seeking and strengthening - Reinforcement of goals, plans, strategies - Expanding the support for change – tools, relationships, services, resources - Termination of helping relationship |
